# Supplementary material for: Bias in the perceived prevalence of open defecation: Evidence from Bihar, India
Source: PLoS One. 2020 Sep 11;15(9):e0238627. doi: 10.1371/journal.pone.0238627 (PMC7485860; doi:10.1371/journal.pone.0238627)
Supplement: S1 File — (DOCX) [file pone.0238627.s001.docx]

**S1 File. Supplemental details of measurements and analysis**

**Qualitative test**

We qualitatively tested different survey item framings, such as “Out of ten people in your community...”, “Out of hundred people in your community...”, “ What percent of people in your community...”, and “Out of these 10 tokens, move how many you think...” to assess numeric comprehension among both male and female respondents (n=25) from similar communities. “Out of ten people in your community” was relatively easy for respondents to understand for the relevant items. We piloted the translated questionnaire with 142 respondents similar to our study population.

**Robustness check varying exclusion criteria**

We found a small proportion of respondents reported that 10 out of 10 community members both use a toilet and defecate in the open. This might suggest they did not comprehend the prevalence questions. We calculate the discrepancy, which is the difference between the perceived prevalence of defecation and the perceived prevalence of not using a toilet. We use the threshold at 5 as our exclusion criteria in the main text. To further test whether the robustness of our finding is sensitive to the exclusion criteria, we conducted a robust test varying the exclusion criteria threshold from 5 to 10. The result suggested that the regression results were robust at different exclusion criteria thresholds, and the result is presented in Table S1.

**Table S1. Robustness check varying exclusion criteria**

|  | Bias in the perceived prevalence | | | | | | | | | |
| --- | --- | --- | --- | --- | --- | --- | --- | --- | --- | --- |
|  |  | | | | | | | | | |
|  | Coef. (CI) | | | | | | | | | |
|  | Threshold >=1 | Threshold >=2 | Threshold >=3 | Threshold >=4 | Threshold >=5 | Threshold >=6 | Threshold >=7 | Threshold >=8 | Threshold >=9 | Threshold >=10 |
| **Inconsistent Users** | **-0.11***** | **-0.11***** | **-0.10***** | **-0.10***** | **-0.10***** | **-0.11***** | **-0.10***** | **-0.10***** | **-0.10***** | **-0.08***** |
|  | (-0.13, -0.08) | (-0.13, -0.08) | (-0.13, -0.08) | (-0.13, -0.08) | (-0.13, -0.08) | (-0.14, -0.09) | (-0.13, -0.08) | (-0.13, -0.08) | (-0.13, -0.08) | (-0.11, -0.05) |
|  |  |  |  |  |  |  |  |  |  |  |
| **Consistent Users** | **-0.20***** | **-0.21***** | **-0.21***** | **-0.21***** | **-0.21***** | **-0.21***** | **-0.21***** | **-0.21***** | **-0.21***** | **-0.22***** |
|  | (-0.23, -0.18) | (-0.23, -0.18) | (-0.23, -0.18) | (-0.23, -0.19) | (-0.23, -0.19) | (-0.24, -0.19) | (-0.24, -0.19) | (-0.24, -0.19) | (-0.24, -0.19) | (-0.25, -0.20) |
|  |  |  |  |  |  |  |  |  |  |  |
| Men | 0.03*** | 0.03*** | 0.02*** | 0.02** | 0.02** | 0.03*** | 0.02** | 0.02** | 0.02** | 0.03*** |
|  | (0.01, 0.05) | (0.01, 0.05) | (0.01, 0.04) | (0.003, 0.04) | (0.01, 0.04) | (0.01, 0.04) | (0.004, 0.04) | (0.004, 0.04) | (0.003, 0.04) | (0.01, 0.05) |
|  |  |  |  |  |  |  |  |  |  |  |
| Primary | -0.03** | -0.03** | -0.03*** | -0.03*** | -0.03*** | -0.03** | -0.03** | -0.03** | -0.03** | -0.03** |
|  | (-0.05, -0.01) | (-0.05, -0.01) | (-0.05, -0.01) | (-0.05, -0.01) | (-0.05, -0.01) | (-0.05, -0.01) | (-0.05, -0.003) | (-0.05, -0.003) | (-0.05, -0.01) | (-0.05, -0.01) |
|  |  |  |  |  |  |  |  |  |  |  |
| Secondary | -0.03* | -0.03* | -0.03* | -0.03* | -0.03** | -0.03* | -0.03* | -0.03* | -0.03* | -0.04** |
|  | (-0.06, 0.003) | (-0.06, 0.001) | (-0.06, 0.002) | (-0.06, 0.0005) | (-0.06, -0.001) | (-0.06, 0.001) | (-0.06, 0.004) | (-0.06, 0.004) | (-0.06, 0.005) | (-0.08, -0.01) |
|  |  |  |  |  |  |  |  |  |  |  |
| High school | -0.06*** | -0.06*** | -0.06*** | -0.06*** | -0.05*** | -0.05*** | -0.05*** | -0.05*** | -0.05*** | -0.06*** |
|  | (-0.09, -0.03) | (-0.09, -0.03) | (-0.09, -0.02) | (-0.09, -0.02) | (-0.09, -0.02) | (-0.08, -0.02) | (-0.08, -0.01) | (-0.08, -0.02) | (-0.08, -0.02) | (-0.10, -0.03) |
|  |  |  |  |  |  |  |  |  |  |  |
| College and above | -0.02 | -0.03 | -0.03 | -0.04* | -0.04** | -0.04** | -0.03 | -0.03* | -0.04* | -0.05** |
|  | (-0.06, 0.02) | (-0.07, 0.01) | (-0.07, 0.01) | (-0.07, 0.001) | (-0.08, -0.004) | (-0.08, -0.002) | (-0.07, 0.01) | (-0.07, 0.01) | (-0.08, 0.001) | (-0.09, -0.01) |
|  |  |  |  |  |  |  |  |  |  |  |
| Age | -0.0004 | -0.0004 | -0.0004 | -0.0004 | -0.0005 | -0.0004 | -0.0004 | -0.0004 | -0.0003 | -0.0005 |
|  | (-0.001, 0.0003) | (-0.001, 0.0003) | (-0.001, 0.0002) | (-0.001, 0.0002) | (-0.001, 0.0002) | (-0.001, 0.0002) | (-0.001, 0.0003) | (-0.001, 0.0003) | (-0.001, 0.0003) | (-0.001, 0.0002) |
|  |  |  |  |  |  |  |  |  |  |  |
| Hindu SC | 0.14*** | 0.13*** | 0.14*** | 0.14*** | 0.13*** | 0.13*** | 0.13*** | 0.13*** | 0.12*** | 0.11*** |
|  | (0.09, 0.18) | (0.09, 0.17) | (0.09, 0.18) | (0.10, 0.18) | (0.09, 0.17) | (0.09, 0.18) | (0.08, 0.17) | (0.08, 0.17) | (0.08, 0.16) | (0.07, 0.16) |
|  |  |  |  |  |  |  |  |  |  |  |
| Muslim | 0.09*** | 0.09*** | 0.10*** | 0.10*** | 0.10*** | 0.10*** | 0.10*** | 0.10*** | 0.09*** | 0.09*** |
|  | (0.05, 0.14) | (0.05, 0.14) | (0.06, 0.14) | (0.06, 0.15) | (0.06, 0.15) | (0.06, 0.15) | (0.05, 0.14) | (0.05, 0.14) | (0.05, 0.14) | (0.04, 0.13) |
|  |  |  |  |  |  |  |  |  |  |  |
| Others | 0.08*** | 0.07*** | 0.08*** | 0.08*** | 0.08*** | 0.08*** | 0.07*** | 0.07*** | 0.07*** | 0.06*** |
|  | (0.03, 0.12) | (0.03, 0.11) | (0.04, 0.11) | (0.04, 0.12) | (0.04, 0.11) | (0.04, 0.12) | (0.03, 0.11) | (0.03, 0.11) | (0.03, 0.10) | (0.02, 0.10) |
|  |  |  |  |  |  |  |  |  |  |  |
| Low SES | -0.01 | -0.02 | -0.01 | -0.01 | -0.01 | -0.01 | -0.01 | -0.01 | -0.01 | -0.01 |
|  | (-0.04, 0.01) | (-0.04, 0.01) | (-0.03, 0.01) | (-0.03, 0.01) | (-0.03, 0.01) | (-0.03, 0.01) | (-0.03, 0.01) | (-0.03, 0.01) | (-0.03, 0.01) | (-0.03, 0.02) |
|  |  |  |  |  |  |  |  |  |  |  |
| Medium SES | -0.04*** | -0.04** | -0.04*** | -0.04** | -0.04*** | -0.04** | -0.04*** | -0.04*** | -0.03** | -0.03* |
|  | (-0.07, -0.01) | (-0.06, -0.01) | (-0.07, -0.01) | (-0.06, -0.01) | (-0.06, -0.01) | (-0.06, -0.01) | (-0.07, -0.01) | (-0.07, -0.01) | (-0.06, -0.01) | (-0.06, 0.0001) |
|  |  |  |  |  |  |  |  |  |  |  |
| High SES | -0.04** | -0.04** | -0.04** | -0.04** | -0.04** | -0.04** | -0.04** | -0.04** | -0.05** | -0.001 |
|  | (-0.08, -0.003) | (-0.08, -0.002) | (-0.08, -0.01) | (-0.08, -0.01) | (-0.08, -0.01) | (-0.08, -0.01) | (-0.08, -0.01) | (-0.08, -0.01) | (-0.08, -0.01) | (-0.04, 0.04) |
|  | | | | | | | | | | |
| **Observations** | **2,131** | **2,257** | **2,339** | **2,384** | **2,413** | **2,444** | **2,457** | **2,459** | **2,468** | **2,528** |
| R2 | 0.25 | 0.25 | 0.25 | 0.25 | 0.25 | 0.24 | 0.23 | 0.23 | 0.23 | 0.23 |
| Adjusted R2 | 0.23 | 0.23 | 0.24 | 0.23 | 0.23 | 0.23 | 0.22 | 0.22 | 0.22 | 0.21 |
| Residual Std. Error | 0.20 (df = 2087) | 0.20 (df = 2213) | 0.20 (df = 2295) | 0.20 (df = 2340) | 0.20 (df = 2369) | 0.20 (df = 2400) | 0.20 (df = 2413) | 0.20 (df = 2415) | 0.21 (df = 2424) | 0.23 (df = 2484) |
| F Statistic | 15.90*** (df = 43; 2087) | 16.80*** (df = 43; 2213) | 17.70*** (df = 43; 2295) | 17.90*** (df = 43; 2340) | 18.10*** (df = 43; 2369) | 17.90*** (df = 43; 2400) | 17.10*** (df = 43; 2413) | 17.20*** (df = 43; 2415) | 16.80*** (df = 43; 2424) | 16.90*** (df = 43; 2484) |
|  | | | | | | | | | | |
| Adjusted p-value | p<0.1; p<0.05; p<0.01 | | | | | | | | | |

**Bivariate analysis**

To identify potential demographics confounders, we conducted bivariate analysis on gender, age, social-economic status (SES), and social-religious groups respondents' self-identified with. Bivariate analysis revealed that respondents’ prevalence perception bias significantly differs by their previous week’s defecation patterns: respondents who defecate in the open perceive that open defecation is more common than it actually is in their community, while those who consistently use a toilet perceived open defecation to be less prevalent (F= 79, p <0.001). Men (M=0.013, SD= 0.238) showed more inflated bias in estimating open defecation prevalence than women (M=-0.003 SD=0.22), although this difference is not significant ( t(2328) = -2 , p=0.09). Respondents with higher education had less inflated prevalence perception bias on open defecation (F= 15.4, p <0.001). Similar patterns of inflated prevalence perception bias of open defecation were found among respondents with lower SES (F = 23.7, p <0.001) and self-identified as Hindu scheduled caste, as compared to Hindu Brahmins (F = 33.4, p<0.001). Age was not significantly associated with prevalence perception bias (t(2328)=0.7, p=0.5). Post-hoc analysis of socio-religion groups revealed that other Hindu scheduled castes, on average have higher levels of bias than other Hindu social castes (t=-6.61, p_adj_<0.001). No other significant difference was found between pairs of socio-religion groups.
